# Arthroscopic removal of intraarticular fragments following fracture dislocation of the hip

**DOI:** 10.4103/0019-5413.40263

**Published:** 2008

**Authors:** Vaibhav Bagaria, Vikram Sapre

**Affiliations:** CIIMS Hospital, Bajaj Nagar, Nagpur, India; 1 NIIDAAN Ortho Centre, Nagpur, India

**Keywords:** Hip arthroscopy, intraarticular fragments, posterior dislocation of the hip

## Abstract

We report here a case of posterior dislocation of hip with fracture of posterior lip of acetabulum, with retained fracture fragments after a successful closed reduction. The fractured fragments were removed by arthroscopy of the hip. The technique of hip arthroscopy used in removing the fragments is discussed.

## INTRODUCTION

Hip arthroscopy provides a clear view of the articular surface of the femoral head, the acetabular labrum, the ligamentum teres and synovium. Today it is becoming popular for procedures not only inside the hip joint but also surrounding it. The various intraarticular conditions for which it is used include labral tears, chondral lesions, hip osteoarthritis, ruptured ligamentum teres, osteonecrosis, femoroacetabular impingement, problems after hip resurfacing or arthroplasty, hip instability, synovial conditions. Extraarticular conditions like snapping illiopsoas or tensor fascia lata and trochanteric bursitis can be treated with minimal invasion.^1^ It has also been shown to be an effective procedure for intraarticular foreign body removal.^2^–^4^ The aim of this case report is to discuss the use of hip arthroscopy as an important tool in removing a small bony osteochondral fragment.

## CASE REPORT

**Figure 1:**
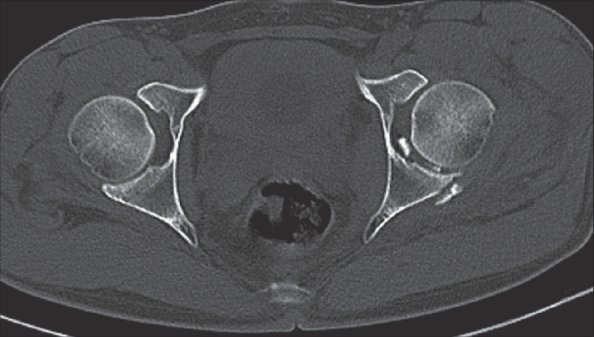
CT scan showing the retained fragments and fracture of the posterior lip of the acetabulum

A 21-year-old male was admitted after a road traffic accident. He had a posterior dislocation of the left hip with a fracture of the posterior acetabular wall (Type III Thompson and Epstein classification) and maxillo-facial fractures. The posterior dislocation was reduced in emergency room under a short general anesthesia using Bigelow reduction maneuver. A follow-up CT scan next morning showed concentric reduction but revealed retained intraarticular fragments. There were two fragments less than 1 cm in size [Figure 1]. Hip arthroscopy was performed to remove the intraarticular fragments.

### Surgical technique

The patient was placed in supine position with the hip to be operated in 10° of flexion, 15° of internal rotation, 10° of lateral tilt and 30° of abduction. An extra wide perineal post was used to minimize the risk of pudendal nerve injury. Traction was applied to break the joint's vacuum seal. Adequate 10 mm of joint distraction for safe surgical instrument clearance was confirmed under image intensifier. Minimal counter traction was also applied to the contralateral leg to reduce the amount of traction necessary on the operative leg.

The anterolateral portal was placed first for the introduction of the arthroscope. An 18-gauge spinal needle was placed under fluoroscopic guidance at the anterior superior corner of the greater trochanter and directed perpendicular to a line drawn from the anterior superior iliac spine (ASIS). Starting as closely as possible to the greater trochanter allows easier passage of the needle under the free margin of the labrum. Once the needle position was confirmed fluoroscopically, a guide wire was introduced and the position of the wire verified under image intensifier. The needle was then removed and a cannulated trocar introduced over the wire after adequate skin incision. The blunt end of the trocar was used to create a tract upto the capsule and the sharp end was used to pop through the thick capsule. The cannula for the arthroscope was then passed over the trocar and finally the camera introduced after starting the irrigation fluid.

The anterior portal was established next. An 18-gauge needle was placed so that it entered the skin at the intersection of a sagittal line drawn distally from the ASIS and a transverse line drawn across the superior margin of the greater trochanter. The needle was directed 45° cephalad and 30° toward the midline, as recommended by Byrd.^3^,^4^ Once return of outflow through the spinal needle had been established, the wire was introduced and the anterior portal established. This portal proved useful for direct visualization of the superior aspect of the femoral head and served as a working portal for instrumentation. An accessory third posterolateral portal was needed and was placed adjacent to the anterolateral portal in line with it but approximately 2 cm more posteriorly.

**Figure 2:**
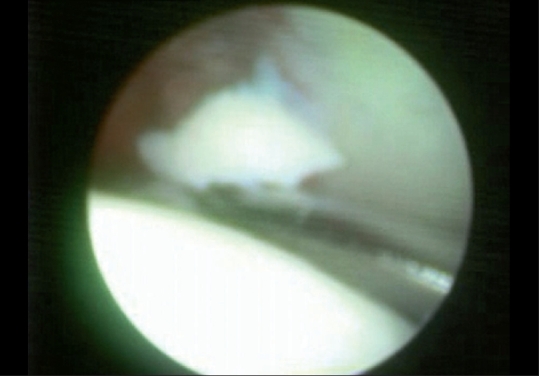
Arthroscopic view of the retained intraarticular fragment

With the arthroscope in the anterolateral portal and shaver in the anterior portal, a grabber was passed through the accessory portal. Both the retained fragments [Figure 2] were removed under vision using the grabber. There was a small unstable partial labral flap tear which was debrided.

Patient was mobilized out of bed after first day, initially partial weight-bearing and then full weight-bearing at one week. He was advised not to place the leg in the adducted and flexed position for the next six weeks. A repeat scan and radiograph was taken and revealed normal joint. At 10-month follow-up, the patient was asymptomatic and the follow-up radiograph and MRI scan did not show any changes of avascular necrosis.

## DISCUSSION

The development of osteoarthritis after dislocation is thought to be due to retained intraarticular fragments or due to incongruent reduction.^5^–^9^ The incidence of such an occurrence has been quite high from 24-54% in different studies.^10^–^13^ Experimental studies have shown that the free cartilaginous particle inside the joint produces chondrolytic enzyme activity thus causing secondary arthrosis.^14^ Hougaard *et al.*, demonstrated that in 15 CT scans of the pelvis after closed reduction of hip dislocations, six patients had intraarticular fragments and another six had minimally displaced fractures of the acetabulum which were not readily visible on routine radiographs.^15^ Open arthrotomy is often the traditional form of treatment for removal of such loose bodies. Furthermore, the surgery often requires a redislocation of hip, leading to an increased chance of development of avascular necrosis.^16^ Minimally invasive techniques like hip arthroscopy can be performed without arthrotomy and redislocation of hip, thereby reducing the chances of avascular necrosis, at the same time permitting the complete visualization of the joint for concentric reduction and the state of the articular cartilage.

There are several studies and case reports that have shown the usefulness of hip arthroscopy in the removal of retained intraarticular fragments after hip dislocation.^17^–^20^ In one of the largest reported series, Yamamoto *et al.*,^20^ performed hip arthrosocopy in 11 cases of hip dislocation and removed intraarticular fragments in nine cases, some of which did not even have any evidence of the same on CT scans. In our study, plain radiographs did not show presence of any intraarticular loose fragments, but CT scan clearly delineated the presence of the same. The sagittal and coronally reconstructed images were especially helpful in this regard. The use of arthrosocopic technique for this particular case offered previously described several advantages over open procedure including diminished blood loss, less disruption of capsule-ligamentous structures, reduced potential for neurovascular damage, decreased recovery time and smaller incisions.

The procedure has its own share of complications, while about 5% of people report worsening of the symptoms after the procedure, a few complications related to procedure may also arise. These are generally related to the distraction of the hip or portal creation and range from 0.5-5%. Most frequently encountered problems are transient or permanent nerve palsies (sciatic, femoral, pudendal or lateral cutaneous), labral or chondral damage and fluid extravasations. Pressure necrosis around the area of perineal post application can occur from excessive traction. The conditions that don't allow safe access like heterotopic ossification, advanced osteoarthritis, protrusio and ankylosis are considered relative contraindications. However, the incidences of serious complications are few.^21^

## CONCLUSION

The use of arthroscopy of the hip in removing loose fracture fragments following posterior dislocation of hip prevents complications associated with open surgery, offers diagnostic and therapeutic benefits with minimal invasiveness and shorter recovery periods than for open procedures.
